# The inverse relationship between the non-high-density lipoprotein cholesterol to high-density lipoprotein cholesterol ratio and testosterone in adult males in the United States: a cross-sectional study based on the NHANES database

**DOI:** 10.3389/fendo.2025.1478124

**Published:** 2025-03-14

**Authors:** Yangyang Mei, Yiming Chen, Xiaogang Wang, Renfang Xu, Rui Xu, Xingliang Feng

**Affiliations:** ^1^ Department of Urology, Jiangyin Hospital Affiliated to Nantong University, Jiangyin, Jiangsu, China; ^2^ Department of Urology, The First People’s Hospital of Changzhou, Changzhou, Jiangsu, China; ^3^ Department of Urology, The Third Affiliated Hospital of Soochow University, Changzhou, Jiangsu, China; ^4^ Department of Rehabilitation Medicine, Affiliated Jinhua Hospital, Zhejiang University School of Medicine, Jinhua Municipal Central Hospital, Jinhua, Zhejiang, China

**Keywords:** testosterone, NHHR, lipid metabolism, testosterone deficiency, NHANES

## Abstract

**Background:**

Testosterone is a crucial hormone for male health, influencing metabolism, cardiovascular function, bone density, and cognitive abilities. Elevated non-HDL cholesterol to HDL cholesterol ratio (NHHR) has been implicated in lipid metabolism disorders, which may adversely affect testosterone levels. This study investigates the association between NHHR and testosterone levels in adult males, utilizing data from the National Health and Nutrition Examination Survey (NHANES).

**Methods:**

This cross-sectional study analyzed data from 2,859 adult males from the NHANES cycles 2011-2016. Total testosterone levels were measured using isotope dilution liquid chromatography-tandem mass spectrometry (ID-LC-MS/MS). NHHR was calculated and analyzed as both a continuous variable and in quartiles. Multivariable linear and logistic regression models, adjusted for demographic, biochemical, lifestyle factors, and medical comorbidities, were used to assess the relationship between NHHR and total testosterone levels and the risk of testosterone deficiency (TD).

**Results:**

Higher NHHR was significantly associated with lower total testosterone levels and increased risk of TD. In fully adjusted models, each unit increase in NHHR was associated with a decrease in total testosterone levels (β = -16.31, 95% CI: -26.58 to -6.04, P = 0.003) and an increased risk of TD (OR = 1.24, 95% CI: 1.07 to 1.44, P = 0.01). When NHHR was analyzed in quartiles, participants in the highest quartile (Q4) had significantly lower testosterone levels (β = -54.98, 95% CI: -86.21 to -23.74, P = 0.001) and a higher risk of TD (OR = 2.04, 95% CI: 1.20 to 3.49, P = 0.01) compared to those in the lowest quartile (Q1). Subgroup analyses confirmed these findings across different age groups, BMI categories, smoking status, and presence of comorbidities. Smooth curve fitting demonstrated a linear relationship among them.

**Conclusion:**

Our study is the first to identify a significant association between elevated NHHR and both reduced total testosterone levels and increased risk of TD in a large, representative sample of adult American males. These findings suggest that NHHR could serve as a valuable marker for early identification of individuals at risk for testosterone decline and TD, enabling timely and targeted clinical interventions.

## 1 Introduction

Testosterone is an indispensable hormone for males, primarily produced by the Leydig cells in the testes and slightly by the adrenal glands, regulated by the negative feedback mechanism of the hypothalamic-pituitary-gonadal axis (HPGA) (1). Beyond its crucial role in sexual and reproductive functions, testosterone significantly impacts metabolism, cardiovascular health, bone density, and cognitive function (2, 3). Correspondingly, insufficient serum testosterone levels in males can result in reduced libido, erectile dysfunction, while also exacerbating metabolic disorders and cardiovascular damage, which is commonly referred to as testosterone deficiency syndrome or male hypogonadism (4, 5). Notably, testosterone deficiency (TD) is a prevalent medical condition affecting American men, with estimates suggesting that 20% to 50% of males have low testosterone levels and nearly 500,000 new cases diagnosed annually (6). The decline in testosterone levels is closely associated with aging; however, metabolic syndrome factors such as obesity, hypertension, and hyperglycemia also significantly increase its prevalence (7). As age advances and lifestyle changes, the incidence of TD is on the rise, posing a significant challenge to public health. This makes it particularly important to identify potential risk factors affecting testosterone levels and implement timely interventions.

Emerging evidence suggests that lipid metabolism disorders can lead to a decline in testosterone levels, which correspondingly increases the risk of TD. Specifically, lipid disorders can directly reduce testosterone production by testicular Leydig cells (8). Additionally, clinical studies have demonstrated a significant relationship between lipid metabolism disorders and testosterone levels, which show that elevated LDL cholesterol and total cholesterol (TC) levels, as well as decreased HDL cholesterol levels, are associated with a decline in testosterone levels (9). Moreover, the decline in testosterone levels is further linearly correlated with these lipid profile changes (10). This bidirectional vicious cycle underscores the importance of investigating the impact of lipid metabolism on testosterone levels and the risk of TD. Recently, the ratio of non-HDL-c to HDL-c (NHHR) has been increasingly recognized as a comprehensive and innovative marker for evaluating atherosclerotic lipid composition (11). Existing studies have shown that NHHR closely correlates with its relevance and predictive value for various diseases. For instance, research by Hong et al. has demonstrated a significant association between NHHR and the occurrence and recurrence of kidney stones, revealing a positive relationship between elevated NHHR and the risk of kidney stones (12). Furthermore, studies have shown that NHHR has higher diagnostic and prognostic value for insulin resistance and metabolic syndrome compared to traditional lipid markers (13). Given the substantial evidence linking lipid metabolism disorders and testosterone levels, exploring the relationship between NHHR and male testosterone levels holds significant scientific value, as it still remains an uncharted area of research to date.

Therefore, the primary aim of this study was to investigate the relationship between NHHR and testosterone levels using data from the National Health and Nutrition Examination Survey (NHANES). By elucidating the effects of lipid metabolism on male reproductive health, this research seeks to deepen our understanding and pave the way for improved strategies to mitigate the risk of TD. Such insights could foster the development of specific preventive and therapeutic approaches, ultimately enhancing patient care and management for conditions associated with TD and related metabolic disorders.

## 2 Materials and methods

### 2.1 Data source and study population

This study utilized data from the NHANES, a program conducted by the Centers for Disease Control and Prevention (CDC). NHANES is designed to assess the health and nutritional status of adults and children in the United States through a combination of interviews and physical examinations. The survey is conducted biennially, providing a continuous dataset that is representative of the civilian, non-institutionalized U.S. population. Data collected include demographic, socioeconomic, dietary, and health-related information, as well as laboratory test results, which are gathered using standardized procedures to ensure consistency and accuracy. This study was approved by the National Center for Health Statistics Ethics Review Board, and all participants provided written informed consent.

**Figure 1:**
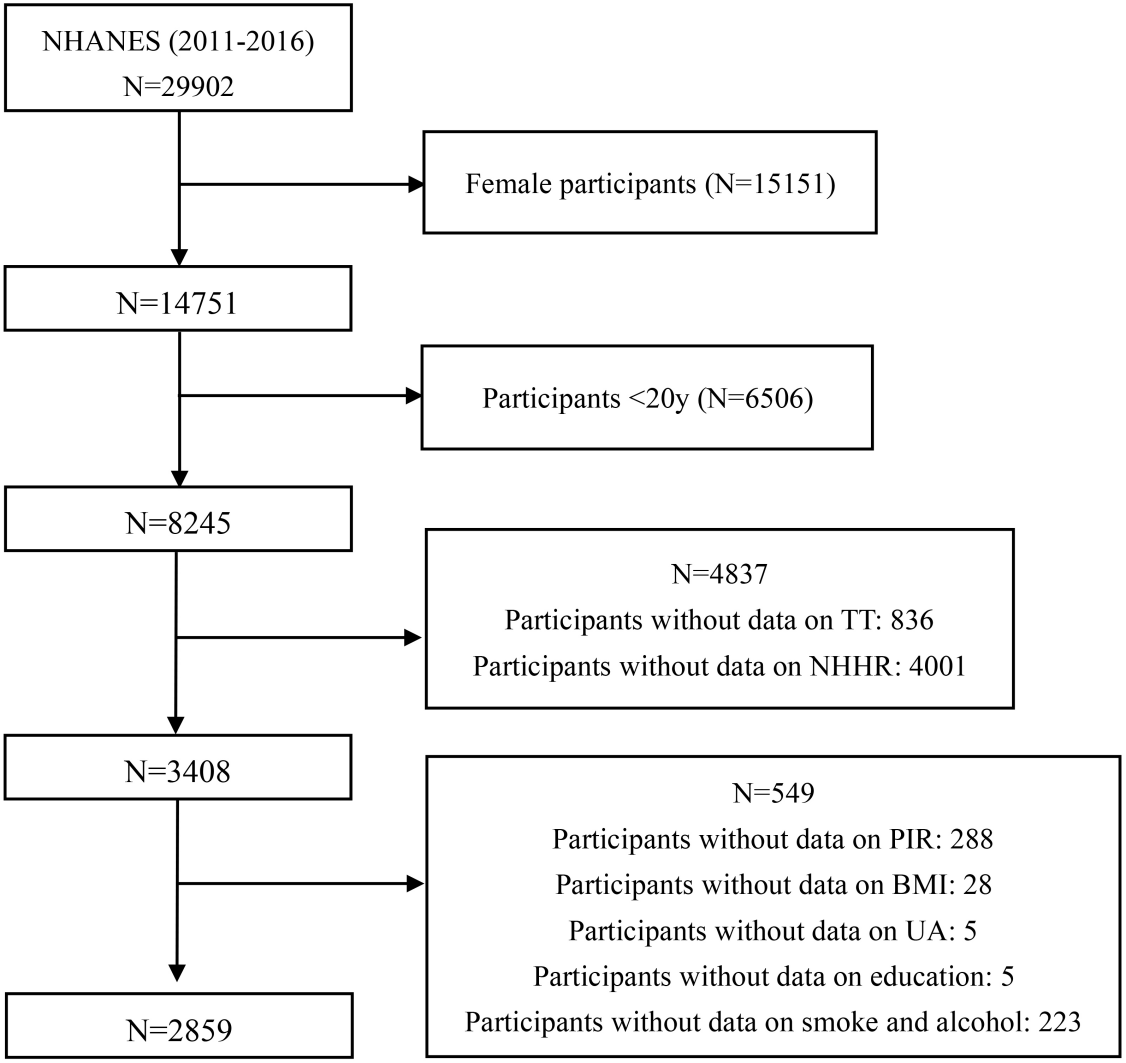
Flowchart of participant selection from the NHANES 2011-2016 cycles. NHANES, National Health and Nutrition Examination Survey; TT, total testosterone levels; NHHR, non-HDL cholesterol to HDL cholesterol ratio; PIR, poverty-income ratio; BMI, body mass index; UA, uric acid.

The NHANES database continuously updates its data in a staggered manner, meaning that at the time of our analysis, the most recent datasets (2022-2023 and preliminary 2024) were not fully available. To ensure completeness and avoid biases introduced by incomplete data, we restricted our analysis to the fully validated NHANES cycles from 2011 to 2016. We included adult males aged 20 years and older who had completed measurements for both testosterone levels and lipid profiles. Additionally, participants needed to have complete data on relevant covariates to be included in the analysis. The inclusion criteria resulted in the exclusion of several groups: 15,151 females, 6,506 individuals under 20 years of age, 836 participants without testosterone measurements, 4,001 without lipid measurements (4,000 missing LDL-c and 1 missing HDL-c), and 549 participants lacking data on covariates. After applying these criteria, the final analytic sample comprised 2,859 individuals from an initial pool of 29,902 participants. Detailed exclusion criteria and participant numbers are presented in **Figure 1**.

### 2.2 Exposure variable measurement

The primary exposure variable in this study was the ratio of non-HDL-c to HDL-c, known as (NHHR) (14). To measure total cholesterol (TC) and HDL-c, and LDL-c, an automated biochemistry analyzer performed enzymatic tests. Specifically, the TC concentrations were determined using Roche Cobas 6000 and Roche Modular P chemical analyzers. Blood samples were drawn from participants after an overnight fast to reduce variability caused by recent food intake. Non-HDL-c was calculated by subtracting HDL-c from TC, providing a measure of all atherogenic lipoproteins present in the blood. The lipid measurements were conducted by certified laboratories using validated assays to ensure accuracy and reliability.

### 2.3 Outcome variable measurement

The primary outcomes in this study were total testosterone levels and the diagnosis of TD. Total testosterone levels were measured using isotope dilution liquid chromatography-tandem mass spectrometry (ID-LC-MS/MS), based on the reference method established by the National Institute for Standards and Technology (NIST). This highly precise technique ensures accurate quantification of testosterone, with a linear detection limit down to 0.75 ng/dL. TD was defined biochemically, given the constraints of the NHANES database, using the threshold recommended by the American Urological Association. According to these guidelines, testosterone deficiency is diagnosed when total testosterone levels are below 300 ng/dL (15). To minimize biological variability, serum specimens were collected in the morning after participants had fasted overnight. The collected samples were then promptly shipped frozen on dry ice for immediate analysis or stored at -70°C for long-term preservation. The Centers for Disease Control and Prevention (CDC) developed the ID-LC-MS/MS method for routine testosterone analysis, ensuring the reliability and validity of the measurements.

### 2.4 Potential covariates

Based on previously published studies, several covariates were included in the analysis to adjust for potential confounding factors that might influence the relationship between NHHR and testosterone levels. These covariates encompassed demographic, biochemical, and lifestyle variables, as well as medical comorbidities. Age was categorized into three groups: 20-40 years, 40-60 years, and > 60 years. Body mass index (BMI) was divided into three categories: <25 kg/m², 25-30 kg/m², and >= 30 kg/m². Additionally, age and BMI were analyzed as a continuous variable to account for its linear relationship with the outcomes. The poverty-income ratio (PIR) was used as an indicator of socioeconomic status, categorized into three groups: <1.3, 1.3 to 3.5, and > 3.5. Race and ethnicity were classified into five categories: Non-Hispanic White, Non-Hispanic Black, Mexican American, Other Hispanic, and Other Race. Educational attainment was grouped into three levels: less than high school, high school graduate, and more than high school. Marital status was classified into solitude and cohabitation.

Biochemical measurements included TC, TG, HDL-c, LDLc, and uric acid (UA). These indicators were measured using standardized laboratory methods to ensure accuracy and reliability. Smoking status was categorized based on lifetime smoking and current smoking behavior: current smokers (who have smoked over 100 cigarettes in their lifetime and currently smoke), former smokers (who have smoked over 100 cigarettes in their lifetime but do not currently smoke), and never smokers (who have smoked fewer than 100 cigarettes in their lifetime). Alcohol consumption was defined as less than 12 drinks per year or 12 or more drinks per year. Medical comorbidities included hypertension, diabetes mellitus (DM), and cardiovascular disease (CVD). Hypertension was defined by a previous diagnosis, self-reported use of antihypertensive medication, or measured blood pressure ≥140/90 mmHg. Diabetes was diagnosed based on a previous diagnosis, use of diabetes medication or insulin, a plasma glucose level ≥200 mg/dL two hours after an oral glucose tolerance test (OGTT), HbA1c ≥6.5%, or a fasting glucose level ≥126 mg/dL. Individuals with impaired fasting glucose (IFG) or impaired glucose tolerance (IGT), indicating abnormal blood glucose levels that do not meet the criteria for DM, were categorized as having borderline DM. CVD was defined by a self-reported history of angina, heart attack, or coronary heart disease.

### 2.5 Statistical analysis

All data analyses were conducted in accordance with the CDC guidelines for NHANES statistical analyses. Given the complex multistage cluster survey design of NHANES, all statistical analyses were appropriately weighted. Initially, we performed descriptive statistics to summarize the study population. Continuous variables were presented as weighted means ± standard errors, and comparisons between groups were made using weighted survey-weighted linear regression. Categorical variables were expressed as weighted percentages, with group comparisons conducted using weighted chi-square tests. To assess the relationship between NHHR and total testosterone levels, multivariable linear regression analyses were employed. The results were reported as beta coefficients with 95% confidence intervals (CIs). Furthermore, multivariable logistic regression analyses were conducted to evaluate the association between NHHR and the risk of TD, with results expressed as odds ratios (ORs) and 95% CIs. In all regression analyses, NHHR was treated both as a continuous variable and as quartiles. Three models were constructed for the regression analyses: Model 1 included no adjustments; Model 2 adjusted for demographic variables, including age, race/ethnicity, marital status, education, and PIR; Model 3 further adjusted for additional covariates, including BMI, smoking status, alcohol consumption, hypertension, DM, CVD, UA, and TC.

Subsequently, we conducted subgroup analyses to explore the stability of the relationship between NHHR and both total testosterone levels and the risk of TD across different populations. Subgroups included age, BMI, smoking status, DM, hypertension, and CVD. Similar to the main regression analyses, NHHR was treated both as a continuous variable and as quartiles, and outcomes included total testosterone levels and the risk of TD. Interaction tests were performed to assess heterogeneity between subgroups. To visualize the potential nonlinear relationship between NHHR and the outcomes, smooth curve fitting and generalized additive models were utilized. This approach allowed for the identification of any nonlinear associations. A two-sided p-value of less than 0.05 was considered statistically significant. To address the risk of Type I errors in subgroup analyses due to multiple comparisons, we employed the Bonferroni correction. The significance threshold was adjusted by dividing the original p-value of 0.05 by the number of subgroups being compared. For example, when comparing three subgroups, statistical significance was defined as p < 0.017. This adjustment helps minimize the risk of false positives and strengthens the reliability of the results. All statistical analyses were performed using EmpowerStats (http://www.empowerstats.com, X&Y Solutions, Inc.) and the statistical software packages R (http://www.R-project.org; The R Foundation).

## 3 Results

### 3.1 Demographic characteristics of study participants

A total of 2,859 participants were included in the study. The mean age of the participants was 47.76 ± 0.41 years, and the mean BMI was 28.92 ± 0.16 kg/m². The mean total testosterone level was 452.47 ± 5.12 ng/dL, with a prevalence of TD of 21.0% (601 out of 2,859 participants). The mean NHHR was 3.37 ± 0.08 among participants with TD, which was significantly higher than the mean NHHR of 2.95 ± 0.03 observed in those with normal testosterone levels. Participants with TD were generally older and had a higher BMI compared to those without TD. Additionally, there were significant differences in smoking and alcohol consumption habits, as well as the prevalence of medical comorbidities between the two groups. Specifically, participants with TD had higher rates of CVD, DM, and hypertension. Moreover, significant differences were also observed between the two groups in terms of UA, HDL-c, and TG. More detailed results are presented in **Table 1**.

**Table 1:** Weighted baseline characteristics of study participants.

Characteristics	Total	Without TD	With TD	P value
Participants number	2859	2258	601	
Age, years	47.76 ± 0.41	46.77 ± 0.55	51.80 ± 0.74	< 0.0001
BMI, kg/m^2^	28.92 ± 0.16	27.86 ± 0.15	33.28 ± 0.48	< 0.0001
TC, mg/dl	186.66 ± 1.08	187.19 ± 1.12	184.50 ± 2.38	0.28
TG, mg/dl	122.72 ± 2.04	115.71 ± 2.07	151.41 ± 4.12	< 0.0001
HDL, mg/dl	49.24 ± 0.40	50.44 ± 0.48	44.31 ± 0.60	< 0.0001
LDL, mg/dl	112.88 ± 0.85	113.60 ± 0.93	109.90 ± 2.08	0.11
Non-HDL	137.43 ± 1.09	136.75 ± 1.13	140.19 ± 2.41	0.18
NHHR	3.03 ± 0.03	2.95 ± 0.03	3.37 ± 0.08	< 0.0001
Total testosterone, ng/dl	452.47 ± 5.12	508.66 ± 5.81	222.43 ± 3.26	< 0.0001
UA, mg/dl	6.10 ± 0.03	5.99 ± 0.03	6.54 ± 0.07	< 0.0001
Age group, %				< 0.001
20-40y	34.90	37.40	24.66	
40-60y	37.61	36.77	41.07	
≥60y	27.49	25.83	34.27	
BMI, %				< 0.0001
Normal (<25 kg/m^2^)	26.54	30.43	10.63	
Overweight (25-30 kg/m^2^)	38.03	40.17	29.27	
Obese (≥30 kg/m^2^)	35.43	29.40	60.09	
PIR, %				0.86
<1.3	20.92	20.99	20.67	
1.3-3.5	35.10	34.80	36.31	
>=3.5	43.98	44.21	43.02	
Race, %				0.48
Non-Hispanic White	70.30	69.63	73.04	
Non-Hispanic Black	8.18	8.32	7.62	
Mexican American	8.30	8.57	7.20	
Other Hispanic	6.00	6.03	5.88	
Other Race	7.22	7.45	6.26	
Education, %				0.93
Less than high school	15.77	15.62	16.38	
High school	22.59	22.69	22.17	
More than high school	61.64	61.69	61.45	
Marital status, %				0.10
Solitude	32.55	33.51	28.66	
Cohabitation	67.45	66.49	71.34	
Smoke, %				< 0.0001
Never	48.00	49.23	42.98	
Former	30.82	28.24	41.40	
Current	21.18	22.53	15.63	
Alcohol, %				< 0.001
No	21.20	19.06	29.94	
Yes	78.80	80.94	70.06	
Hypertension, %				< 0.0001
No	59.58	62.60	47.23	
Yes	40.42	37.40	52.77	
Diabetes, %				< 0.0001
No	62.69	67.89	41.41	
Prediabetes	20.03	18.62	25.82	
Yes	17.28	13.50	32.76	
CVD, %				< 0.0001
No	89.42	91.03	82.84	
Yes	10.58	8.97	17.16	

TD, Testosterone Deficiency; BMI, Body Mass Index; TC, Total Cholesterol; TG, Triglycerides; HDL, High-Density Lipoprotein Cholesterol; LDL, Low-Density Lipoprotein Cholesterol; NHHR, Non-HDL Cholesterol to HDL Cholesterol Ratio; UA, Uric Acid; PIR, Poverty-Income Ratio; CVD, Cardiovascular Disease.

Data are presented as weighted means ± standard errors or percentages; Statistical significance was determined using weighted survey-weighted linear regression for continuous variables and weighted chi-square tests for categorical variables.

### 3.2 Regression analysis and smooth curve fitting

**Figure 2:**
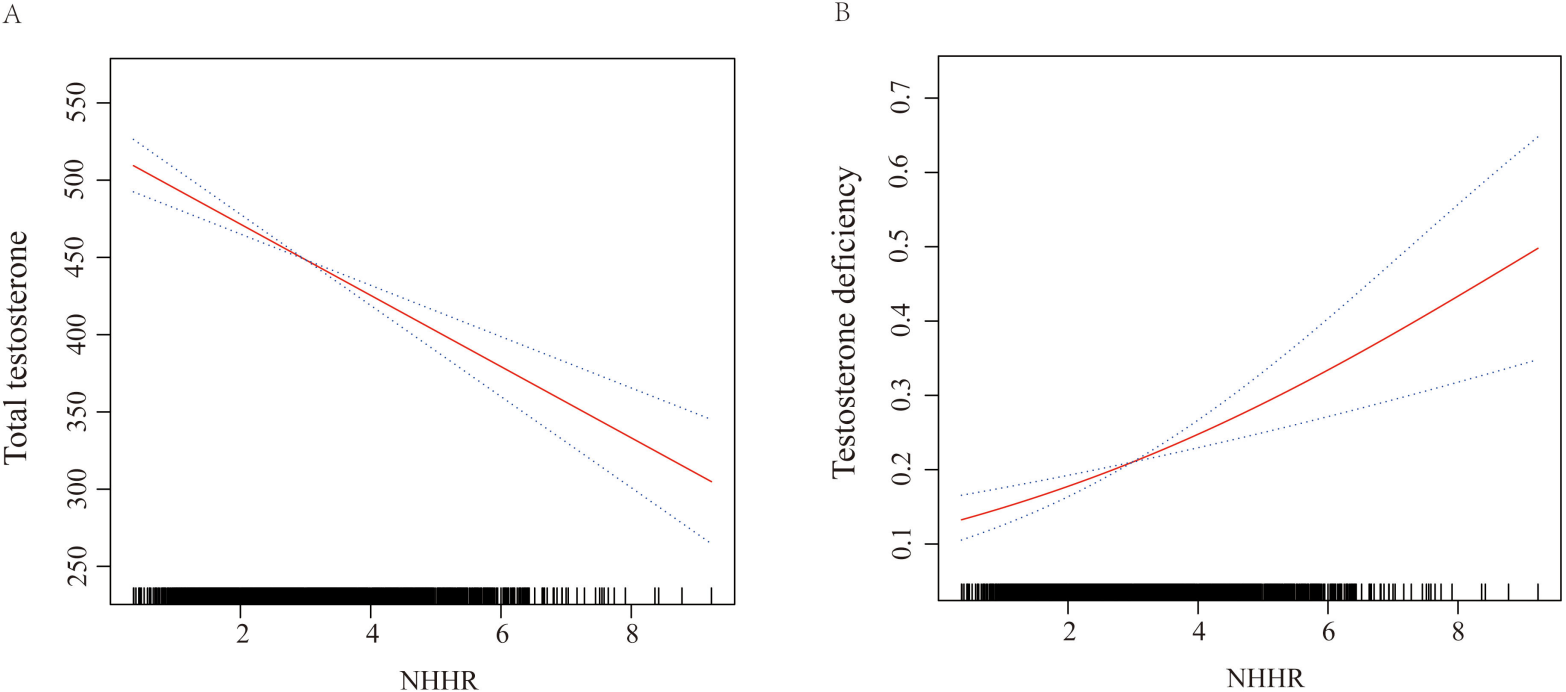
Graphics of smooth curve fittings illustrating the relationship between continuous NHHR and **(A)** total testosterone levels, and **(B)** risk of testosterone deficiency (TD). The odds ratios (ORs), depicted by solid lines, were adjusted for age, race/ethnicity, marital status, education level, poverty-income ratio (PIR), body mass index (BMI), smoking status, alcohol consumption, hypertension, diabetes mellitus (DM), cardiovascular disease (CVD), uric acid (UA), and total cholesterol (TC). Corresponding 95% confidence intervals (CIs) are represented by black dashed lines.

Our primary outcome variable was total testosterone level, while the primary exposure variable was continuous NHHR. The analysis revealed that an increase in NHHR was consistently associated with a decline in total testosterone levels across all models. Specifically, in Model 1, the relationship was quantified as β = -27.77 (95% CI: -34.51 to -21.02, P < 0.0001). This inverse association remained robust in Model 2, with β = -28.56 (95% CI: -35.39 to -21.72, P < 0.0001). Even after full adjustment in Model 3, the relationship persisted, though slightly attenuated, with β = -16.31 (95% CI: -26.58 to -6.04, P = 0.003). When NHHR was categorized into quartiles, using the lowest quartile (Q1) as the reference group, Model 3 demonstrated that participants in the highest quartile (Q4) had significantly lower testosterone levels, with β = -54.98 (95% CI: -86.21 to -23.74, P = 0.001), and a significant trend test. This trend was consistent across Model 1 and Model 2 as well. Further analysis using TD as the outcome variable reinforced these findings. In the fully adjusted Model 3, a continuous increase in NHHR was associated with an elevated risk of TD (OR = 1.24, 95% CI: 1.07 to 1.44, P = 0.01). Comparing quartiles, participants in Q4 had a significantly higher risk of TD compared to those in Q1 (OR = 2.04, 95% CI: 1.20 to 3.49, P = 0.01). These significant associations were consistently observed in Model 1 and Model 2 as well. Detailed results are presented in **Table 2**. The smooth curve fitting, conducted using Model 3, illustrated a clear negative linear relationship between NHHR and total testosterone levels, and a positive linear relationship with the risk of TD. These relationships are visually depicted in **Figure 2A, B**, providing a clear and intuitive presentation of the data.

**Table 2:** Weighted linear and logistic regression analysis of NHHR with total testosterone Levels and testosterone deficiency.

	Model 1	Model 2	Model 3
Total testosterone (ng/dl)-β (95%CI) p-value			
Continuous	-27.77(-34.51, -21.02), P<0.0001	-28.56(-35.39, -21.72), P<0.0001	-16.31(-26.58, -6.04), 0.003
Quartile 1	Reference	Reference	Reference
Quartile 2	-33.78(-55.29, -12.27), 0.003	-32.17(-53.51, -10.84), 0.004	-17.31(-39.33, 4.71), 0.12
Quartile 3	-53.54(-78.99, -28.09), P<0.001	-52.6(-78.61, -26.59), P<0.001	-14.48(-40.22, 11.26), 0.26
Quartile 4	-94.46(-119.03, -69.90), P<0.0001	-95.34(-119.32, -71.37), P<0.0001	-54.98(-86.21, -23.74), 0.001
P for trend	<0.0001	<0.0001	0.003
Testosterone deficiency-OR (95% CI) p-value			
Continuous	1.28(1.17,1.41), P<0.0001	1.32(1.19,1.45), P<0.0001	1.24(1.07,1.44), 0.01
Quartile 1	Reference	Reference	Reference
Quartile 2	1.60(1.14,2.25), 0.01	1.67(1.18,2.38), 0.01	1.43(0.96,2.12), 0.07
Quartile 3	1.80(1.24,2.61), 0.003	1.94(1.32,2.83), 0.001	1.30(0.82,2.04), 0.25
Quartile 4	2.46(1.73,3.51), P<0.0001	2.73(1.89,3.95), P<0.0001	2.04(1.20,3.49), 0.01
P for trend	<0.0001	<0.0001	0.013

Model 1: Unadjusted.

Model 2: Adjusted for demographic variables including age, race/ethnicity, marital status, education level, and PIR.

Model 3: Further adjusted for additional covariates including BMI, smoking status, alcohol consumption, hypertension, DM, CVD, UA, and TC.

TD, Testosterone Deficiency; BMI, Body Mass Index; TC, Total Cholesterol; NHHR, Non-HDL Cholesterol to HDL Cholesterol Ratio; UA, Uric Acid; PIR, Poverty-Income Ratio; CVD, Cardiovascular Disease; DM, diabetes mellitus; OR, Odds Ratio; 95% CI, 95% Confidence Interval.

### 3.3 Subgroup analysis

In the subgroup analyses, we examined the relationship between continuous NHHR and the outcomes of total testosterone levels and TD, adjusting for all variables included in Model 3 except for the grouping variable itself. For age subgroups, the analysis demonstrated a significant inverse relationship between NHHR and total testosterone levels in participants aged 20-40 years (β = -24.49, 95% CI: -38.01 to -10.96). However, no significant association was observed between NHHR and TD in any age subgroup. Regarding BMI subgroups, the relationship between NHHR and total testosterone levels, as well as the risk of TD, was significant only in participants with a BMI >30 kg/m^2^. Specifically, the β coefficient for total testosterone was -26.56 (95% CI: -43.55 to -9.58), and the OR for TD was 1.54 (95% CI: 1.23 to 1.93). In the smoking status subgroups, no significant associations were found between NHHR and either total testosterone levels or TD among current smokers. For participants with and without DM and CVD, significant associations between NHHR and both outcomes were only observed in those without these comorbidities. Conversely, for hypertension, significant associations were found regardless of the presence or absence of the condition. Detailed results of the subgroup analyses are presented in **Table 3**, with no interaction effects detected across all subgroups.

**Table 3:** Weighted subgroup analysis of continuous NHHR with total testosterone levels and testosterone deficiency.

Subgroup	β (95%CI)	P value	P for interaction	OR (95%CI)	P value	P for interaction
Age group			0.07			0.42
20-40y	-24.49 (-38.01, -10.96)	**<0.001**		1.21 (0.93, 1.57)	0.15	
40-60y	-11.85 (-25.20, 1.49)	0.08		1.14 (0.91,1.44)	0.25	
>60y	-6.57 (-27.00,13.86)	0.52		1.32 (0.95,1.82)	0.09	
BMI			0.17			0.12
Normal	-5.54 (-23.27, 12.18)	0.53		1.02 (0.71,1.48)	0.90	
Overweight	-13.77 (-27.75, 0.21)	0.05		1.19 (0.94,1.50)	0.14	
Obese	-26.56 (-43.55, -9.58)	**0.003**		1.54 (1.23,1.93)	**<0.001**	
Smoking status			0.87			0.04
Never	-19.15 (-34.21, -4.08)	**0.01**		1.25 (1.00,1.56)	0.05	
Former	-21.7 (-32.64, -10.76)	**<0.001**		1.56 (1.26,1.93)	**<0.001**	
Current	-8.05 (-25.40, 9.31)	0.35		0.90 (0.64,1.27)	0.55	
DM			0.90			0.82
No	-15.21 (-26.54, -3.88)	**0.01**		1.25 (1.06,1.48)	**0.01**	
Borderline	-17.99 (-34.73, -1.26)	0.04		1.13 (0.85,1.50)	0.39	
Yes	-20.35 (-44.42, 3.72)	0.09		1.30 (0.91,1.85)	0.14	
Hypertension			0.63			0.73
No	-16.98 (-28.02, -5.95)	**0.004**		1.22 (1.01,1.47)	0.04	
Yes	-17.59 (-32.07, -3.11)	0.02		1.28 (1.01,1.62)	0.04	
CVD			0.13			0.42
No	-14.56 (-25.16, -3.97)	**0.01**		1.24 (1.08,1.42)	**0.003**	
Yes	-24.21 (-49.18, 0.76)	0.06		1.23 (0.81,1.86)	0.31	

Analyses were conducted in Model 3, with full adjustment for all covariates except the subgroup variable itself.

BMI, Body Mass Index; NHHR, Non-HDL Cholesterol to HDL Cholesterol Ratio; DM, diabetes mellitus; CVD, Cardiovascular Disease; OR, Odds Ratio; 95% CI, 95% Confidence Interval.

The bold values indicate p < 0.05, representing statistical significance.

Subsequently, we analyzed the associations using NHHR quartiles as the exposure while keeping the outcomes as total testosterone levels and TD. The subgroup analyses were similarly adjusted for all variables in Model 3, excluding the grouping variable. Detailed results are presented in **Table 4**. Using Q1 as the reference, the significant associations between NHHR and total testosterone levels were observed in several subgroups: participants aged 20-40 years (β = -98.64, 95% CI: -139.33 to -57.95), those with BMI > 25 kg/m², non-smokers (β = -73.71, 95% CI: -116.67 to -30.75), those without hypertension (β = -55.84, 95% CI: -89.45 to -22.23), and those without DM (β = -59.32, 95% CI: -95.80 to -22.83). For CVD subgroups, significant associations were found regardless of the presence or absence of these conditions. When considering TD as the outcome, significant associations between NHHR and the risk of testosterone deficiency were observed in specific subgroups. These included participants with BMI > 30 kg/m² (OR = 3.79, 95% CI: 1.61 to 8.93), non-smokers (OR = 2.29, 95% CI: 1.24 to 4.25), former smokers (OR = 3.42, 95% CI: 1.54 to 7.57), and those without CVD (OR = 1.89, 95% CI: 1.06 to 3.38). Consistent with the previous analyses, no interaction effects were detected across all subgroups, confirming the robustness and consistency of the observed associations. Significance of the above subgroup analyses were based on Bonferroni-corrected p-values.

**Table 4:** Weighted subgroup analysis of NHHR quartiles with total testosterone levels and testosterone deficiency.

Subgroup	Quartile 1	Quartile 2	Quartile 3	Quartile 4	P for trend	P for interaction
Total testosterone (ng/dl)-β (95%CI)						
Age group						0.06
20-40y	Reference	-38.57 (-69.09, -8.04)	-34.77 (-82.01, 12.47)	-98.64 (-139.33, -57.95)	<0.001	
40-60y	Reference	-33.9 (-70.45, 2.65)	-26.69 (-66.12, 12.75)	-39.74 (-82.58, 3.10)	0.11	
>60y	Reference	23.62 (-26.92, 74.16)	35.87 (-24.86, 96.60)	-19.17 (-84.73, 46.38)	0.93	
BMI						0.60
Normal	Reference	-7.78 (-44.26, 28.71)	8.34 (-38.93, 55.61)	-42.64 (-102.41, 17.13)	0.40	
Overweight	Reference	-23.07 (-64.33, 18.20)	-31.37 (-68.78, 6.05)	-80.54 (-131.87, -29.20)	0.01	
Obese	Reference	-19.76 (-61.54, 22.03)	-27.7 (-66.17, 10.77)	-44.83 (-88.15, -1.50)	0.04	
Smoking status						0.90
Never	Reference	-21.94 (-59.49, 15.61)	-27.93 (-74.29, 18.43)	-73.71 (-116.67, -30.75)	0.004	
Former	Reference	-4.1 (-46.70, 38.49)	-9.26 (-47.51, 28.99)	-51.4 (-89.89, -12.91)	0.01	
Current	Reference	-44.06 (-90.41, 2.30)	-14.28 (-64.61, 36.06)	-40.19 (-94.44, 14.05)	0.25	
DM						0.71
No	Reference	-29.12 (-55.35, -2.90)	-19.69 (-49.18, 9.80)	-59.32 (-95.80, -22.83)	0.005	
Borderline	Reference	4.66 (-39.88, 49.20)	-16.22 (-73.18, 40.73)	-40.23 (-97.65, 17.20)	0.13	
Yes	Reference	11.94 (-52.59, 76.47)	15.18 (-51.24, 81.60)	-48.55 (-132.28, 35.18)	0.31	
Hypertension						0.74
No	Reference	-9.47 (-36.39, 17.45)	-11.89 (-42.28, 18.50)	-55.84 (-89.45, -22.23)	0.004	
Yes	Reference	-34.31 (-70.48, 1.86)	-26.4 (-74.42, 21.62)	-59.91 (-106.44, -13.38)	0.04	
CVD						0.65
No	Reference	-12.51 (-37.83, 12.81)	-10.89 (-40.32, 18.54)	-46.82 (-79.55, -14.08)	0.01	
Yes	Reference	-51.83 (-120.32, 16.66)	-53.62 (-114.70, 7.47)	-110.81 (-188.53, -33.09)	0.01	
Testosterone deficiency-OR (95% CI)						
Age group						0.15
20-40y	Reference	1.37 (0.55,3.42)	1.02 (0.35,3.02)	2.14 (0.71,6.50)	0.20	
40-60y	Reference	2.51 (1.06,5.93)	1.95 (0.87,4.34)	1.87 (0.74,4.72)	0.49	
>60y	Reference	1.12 (0.63,1.99)	1.08 (0.48,2.45)	2.45 (0.89,6.71)	0.14	
BMI						0.36
Normal	Reference	0.79 (0.32,1.94)	1.29 (0.35,4.69)	1.59 (0.52,4.82)	0.48	
Overweight	Reference	1.71 (0.71,4.14)	1.70 (0.80,3.59)	1.88 (0.70,5.02)	0.25	
Obese	Reference	1.69 (0.86,3.33)	1.56 (0.73,3.31)	3.79 (1.61,8.93)	0.004	
Smoking status						0.12
Never	Reference	1.54 (0.90,2.65)	1.63 (0.93,2.85)	2.29 (1.24,4.25)	0.01	
Former	Reference	1.77 (0.93,3.36)	1.86 (0.97,3.55)	3.42 (1.54,7.57)	0.01	
Current	Reference	0.98 (0.55,1.77)	0.50 (0.20,1.29)	0.74 (0.29,1.88)	0.38	
DM						0.90
No	Reference	1.74 (0.99,3.05)	1.41 (0.69,2.89)	2.02 (0.89,4.59)	0.15	
Borderline	Reference	0.97 (0.38,2.47)	1.00 (0.46,2.19)	1.43 (0.53,3.86)	0.41	
Yes	Reference	1.35 (0.62,2.96)	1.45 (0.67,3.12)	2.65 (0.89,7.94)	0.09	
Hypertension						0.97
No	Reference	1.34 (0.74,2.40)	1.29 (0.66,2.54)	1.96 (0.88,4.33)	0.10	
Yes	Reference	1.54 (0.87,2.73)	1.33 (0.71,2.49)	2.23 (1.05,4.73)	0.06	
CVD						0.76
No	Reference	1.37 (0.83,2.25)	1.31 (0.78,2.21)	1.89 (1.06,3.38)	0.03	
Yes	Reference	2.11 (0.62, 7.23)	1.45 (0.46, 4.63)	3.54 (0.91,13.77)	0.12	

Analyses were conducted in Model 3, with full adjustment for all covariates except the subgroup variable itself.

BMI, Body Mass Index; NHHR, Non-HDL Cholesterol to HDL Cholesterol Ratio; DM, diabetes mellitus; CVD, Cardiovascular Disease; OR, Odds Ratio; 95% CI, 95% Confidence Interval.

## 4 Discussion

In this analysis involving 2,859 adult American males, we observed that participants with higher NHHR were more likely to have lower total testosterone levels and a higher risk of TD. These associations remained robust across different population characteristics, including age, BMI, smoking status, and the presence of DM, hypertension, or CVD, as verified by subgroup analyses and interaction tests. Moreover, we found a linear relationship between increasing continuous NHHR and both declining total testosterone levels and increasing risk of TD. Consequently, utilizing NHHR in clinical practice to identify individuals at risk for testosterone decline or deficiency holds substantial potential benefits.

Clinical studies have shown that there is a significant relationship between lipid metabolism disorders and testosterone levels. Research indicates that increased levels of LDL-c) and TC, as well as decreased levels of HDL-c, are associated with a decline in testosterone levels (9). Additionally, the decrease in testosterone levels is linearly correlated with these changes in blood lipids (10). Previous studies have long demonstrated that HDL-C levels are positively correlated with testosterone levels, while very-low-density lipoprotein cholesterol (VLDL-C) levels are negatively correlated with testosterone levels (9, 16). Therefore, a comprehensive assessment of no-HDL-c and HDL-c homeostasis is more conducive to understanding its relationship with testosterone.

NHHR is a newly developed index used to assess atherosclerotic blood lipids (the ratio of non-HDL-C to HDL-C), and it is related to dyslipidemia-related diseases (17). An increase in NHHR may indicate an imbalance in lipid metabolism. Currently, many related studies have shown that NHHR can independently determine the risk of atherosclerosis, insulin resistance and metabolic syndrome, chronic kidney disease, and non-alcoholic fatty liver disease (13, 18–20), outperforming standard lipid parameters in prediction and diagnostic efficacy. Recent research had also reported associations between NHHR and various diseases such as depression, abdominal aortic aneurysm, and diabetes (17, 21, 22). Therefore, in our study, we demonstrated the relationship between NHHR and TD, showing that higher NHHR is associated with lower total testosterone levels and an increased risk of TD. While NHHR offers a novel approach to predicting TD, it is important to consider how it compares with existing diagnostic criteria. Currently, the diagnosis of TD is typically based on serum testosterone levels and the presence of clinical symptoms, with a testosterone level threshold of < 300 ng/dL. In comparison, NHHR integrates both lipid metabolism and inflammation, factors that are strongly associated with testosterone regulation. By providing a more comprehensive view of an individual’s metabolic and inflammatory status, NHHR could serve as a complementary tool for risk stratification, helping to identify individuals at risk for TD earlier, particularly in those with underlying metabolic disturbances. However, further validation in clinical cohorts is needed to fully assess NHHR’s role in clinical practice and its predictive accuracy compared to traditional diagnostic criteria.

This study initially investigated the relationship between NHHR and testosterone, and the underlying mechanisms between the two are unclear. HDL-C inhibits the formation of atherosclerotic plaques and has anti-inflammatory, antithrombotic, and antioxidant effects in patients with cardiovascular disease (23). Additionally, HDL-C primarily reduces the oxidation of LDL-C, thereby inhibiting the inflammatory activation of endothelial cells by LDL-C (24). However, a decrease in HDL-C levels can activate inflammatory and oxidative pathways, thereby accelerating the progression of diseases. Under normal physiological conditions, HDL-C can remove endotoxins (LPS) from the bloodstream, inhibit the maturation and activation of macrophages and lymphocytes, and reduce inflammatory responses (25). Additionally, HDL-C has antioxidant properties, which can decrease lipid peroxidation reactions, thus protecting mitochondrial function and energy production (26). On the other hand, high levels of non-HDL-c promote the activation of inflammatory pathways. Elevated levels of pro-inflammatory cytokines such as IL-6, TNF-α, and MCP-1 have been observed in populations with high non-high-density lipoprotein cholesterol (27). Additionally, high levels of non-HDL-c lead to an imbalance between pro-oxidant and antioxidant mechanisms. This imbalance increases the production of reactive oxygen species (ROS), such as superoxide and hydroxyl radicals, which damage cellular components, including lipids, proteins, and DNA (28). Currently, Mets (including obesity, dyslipidemia, hypertension, and insulin resistance (IR)) is a risk factor for testosterone deficiency (29). The activation of inflammation and increased oxidative stress are indeed mechanisms of Mets (30). Moreover, Obesity may increase inflammatory factors, IL-6, IL-8, and IL-1β, thereby increasing IR (31, 32). In IR, various cytokines and inflammatory mediators, particularly CRP, TNF-α, MCP-1, and IL, are upregulated (33, 34). While NHHR is a novel marker, its relevance to endocrine research has not been as extensively explored as traditional lipid markers. The rationale for using NHHR rather than traditional lipid markers such as TC, HDL-C, or LDL-C lies in its ability to integrate lipid metabolism and inflammation, two critical factors involved in testosterone regulation and TD. Traditional lipid markers often fail to capture the systemic inflammation that influences testosterone synthesis, particularly in metabolic syndrome and insulin resistance, where lipid imbalance and inflammation coexist. In contrast, NHHR reflects both triglyceride-glucose interactions, which are closely linked to insulin resistance and systemic inflammation, offering a more comprehensive view of the metabolic environment affecting testosterone regulation. Therefore, an increase in NHHR may indicate a pro-inflammatory and pro-oxidative state associated with the pathogenesis of TD, similar to what is observed in Mets. While this study reports a statistically significant association between NHHR and testosterone levels (e.g., β = -16.31, P = 0.003), we recognize that small absolute changes in testosterone may not always result in clinically significant effects. Even though the decline in testosterone levels observed in this study is statistically significant, it may not reach a threshold that leads to meaningful clinical consequences for individuals. Nonetheless, modest changes in testosterone could still be relevant in populations at risk for TD, where even slight declines may contribute to symptoms like fatigue, reduced libido, or muscle weakness (35). Future studies should aim to determine the clinical thresholds for testosterone decline and incorporate symptom-based measures or quality of life assessments to better understand the true clinical impact of small changes in testosterone.

The observed differences in the strength of the association between NHHR and testosterone levels across different subgroups suggest that the biological mechanisms linking NHHR to testosterone regulation and TD may vary. Factors such as inflammation, insulin resistance, adiposity-related hormones, and age-related changes could contribute to these subgroup-specific effects. However, the current study did not explore these mechanistic differences, and we attempt to discuss the potential mechanisms underlying these differences here. Testosterone levels naturally decline with age, particularly after the age of 40. Older individuals may have a diminished capacity to respond to metabolic or inflammatory insults compared to younger individuals (36). As NHHR is linked to lipid metabolism and systemic inflammation (11), its effect on testosterone may be exacerbated in older adults due to age-related increases in inflammation, insulin resistance, and adiposity (6). Younger individuals, by contrast, may experience greater metabolic flexibility (37), where hormonal pathways, such as the hypothalamic-pituitary-gonadal (HPG) axis, can still counterbalance metabolic disturbances. This could explain the stronger association in younger participants. Elevated BMI (especially in the obese group) is closely linked to increased adiposity, which promotes chronic low-grade inflammation and insulin resistance (38). Higher levels of non-HDL cholesterol in individuals with obesity can further contribute to endothelial dysfunction, increased oxidative stress, and testicular steroidogenesis impairment (39). Obesity-related cytokines (such as TNF-α, IL-6) may inhibit the HPG axis and impair Leydig cell function, further decreasing testosterone secretion. The stronger association between NHHR and testosterone in obese individuals likely reflects this synergistic effect between metabolic disturbances and hormone regulation. Smoking is a well-known risk factor for systemic inflammation and vascular dysfunction (40), which can impact both testosterone production and lipid metabolism. Smokers often have lower HDL-C levels, higher oxidative stress, and a reduced antioxidant capacity, all of which can influence testosterone levels (41). Smokers may also experience altered gonadal function due to nicotine-induced sympathetic nervous system activation (42). The observed stronger association in non-smokers may suggest that the harmful effects of smoking on inflammation and metabolic health mask the relationship between NHHR and testosterone in smokers. DM and CVD are both associated with increased systemic inflammation, oxidative stress, and insulin resistance, all of which are known to affect testosterone production (43, 44). In individuals with DM or CVD, the inflammatory environment may already be so pronounced that further contributions from NHHR (a lipid-related marker of inflammation) might be less detectable. In contrast, individuals without these comorbidities may exhibit more pronounced relationships between NHHR and testosterone, as their metabolic systems may be more responsive to changes in lipid metabolism and inflammatory markers. This could explain why individuals without DM or CVD showed stronger associations. Moreover, although no significant interaction effects were detected across subgroups in this study, we recognize that certain potential interactions, such as those between obesity and lipid metabolism, may warrant further exploration. The absence of significant findings may be due to statistical limitations or the complexity of these interactions, which could require more advanced statistical approaches, such as multilevel modeling or structural equation modeling (SEM). Future studies should explore these potential interactions using more sophisticated models to better understand the subgroup-specific effects and their implications for testosterone regulation.

This study has several notable strengths. First, we are the first to investigate the association between NHHR and testosterone levels using the NHANES database, which ensures high-quality data, representativeness of the population, and ample sample size. Second, our study meticulously adjusted for all potential confounding variables based on previously published literature on factors affecting testosterone levels. This rigorous approach, supplemented by subgroup analyses and restricted cubic spline (RCS) analysis, strengthens the robustness and reliability of our findings. However, several inherent limitations must be considered when interpreting our results. First, given the cross-sectional design of this study, we acknowledge that the observed association between NHHR and testosterone levels cannot establish causality. While we have identified a significant correlation, it is important to recognize that the direction of this relationship remains unclear. Reverse causation—where changes in testosterone could influence NHHR—cannot be excluded. To better understand the causal relationship between NHHR and testosterone levels, future studies should utilize longitudinal or experimental designs that can assess temporal changes and control for potential confounders. Second, the diagnosis of TD in our study was solely based on biochemical measurements of testosterone levels, without considering the symptomatic manifestations of testosterone deficiency syndrome. Third, despite adjusting for multiple metabolic and lifestyle factors, the possibility of residual confounding cannot be ruled out. Unmeasured variables such as dietary habits, physical activity, medication use (e.g., statins, testosterone therapy), and genetic predispositions may influence both NHHR and testosterone levels. Our covariate selection was guided by prior research, balancing comprehensiveness with model stability. However, future studies should incorporate these additional factors to further refine the understanding of these associations. Fourth, while we analyzed NHHR both as a continuous variable and by categorizing it into quartiles, the quartile approach, while useful for clinical interpretation, may reduce statistical power and obscure more subtle associations that a continuous analysis might capture. This categorization could potentially impact the accuracy and precision of the results. Future studies may benefit from using continuous variables or alternative methods to explore finer relationships. Moreover, in this study, testosterone levels were measured using isotope dilution liquid chromatography-tandem mass spectrometry (ID-LC-MS/MS), which is considered the gold standard for steroid hormone quantification due to its high specificity and accuracy. However, this method requires specialized equipment and technical expertise, and variability across laboratories may arise due to differences in calibration standards and assay conditions. While NHANES implements strict quality control measures following CDC and NIST guidelines to standardize testosterone measurements, minor inter-laboratory variations cannot be entirely ruled out. Additionally, Testosterone levels fluctuate due to diurnal variation, stress, and physical activity. NHANES minimizes this by collecting blood samples in the morning, but a single measurement may not fully reflect long-term testosterone status. Future studies should incorporate repeated measurements to enhance reliability. Furthermore, the significant associations observed between NHHR and testosterone levels were primarily seen in specific subgroups, such as younger participants, those with BMI >30 kg/m², non-smokers, and individuals without diabetes or CVD. While these associations are noteworthy, they may not be applicable to all demographic or health-related subgroups. Subgroup-specific effects could be influenced by factors such as age, metabolic status, smoking, and comorbidities, which may alter the relationship between NHHR and TD. These findings should be viewed as hypothesis-generating, and future studies should aim to validate these associations across more diverse and representative populations to assess their broader applicability. Lastly, the exclusion of individuals with missing lipid profile or testosterone measurement data may introduce potential bias and limit the generalizability of our findings, especially to populations with incomplete medical records. Although we used multiple imputation or complete case analysis to handle the missing data, we acknowledge that these methods may not fully eliminate bias. Future research should focus on including populations with more complete data or exploring advanced imputation methods to improve the generalizability and robustness of the findings. While this study demonstrates a significant association between NHHR and testosterone levels, we recognize that this relationship may be influenced by several confounding factors, including systemic inflammation, insulin resistance, adiposity-related hormones, and lifestyle factors. These factors may contribute to TD independently of NHHR. Furthermore, alternative explanations, such as the role of lipid metabolism, endothelial dysfunction, and vascular health, may also account for the observed association. We acknowledge that the observed associations should be interpreted with caution and that NHHR’s potential as a predictive tool for TD needs further exploration in future studies, with a more thorough control for confounders. Therefore, prospective studies with larger sample sizes and the inclusion of more comprehensive covariates are essential to confirm our results and further elucidate the relationship between NHHR and testosterone levels.

## 5 Conclusion

In conclusion, our comprehensive analysis reveals a significant association between elevated NHHR and both reduced total testosterone levels and an increased risk of TD. This finding highlights the potential role of lipid metabolism in regulating male androgen levels and suggests that NHHR could be a promising biomarker for identifying individuals at risk for declining testosterone levels and TD. However, it is important to note that the cross-sectional nature of the study limits our ability to establish causality and assess the long-term clinical implications of NHHR. While these findings are promising, further validation through longitudinal studies and clinical trials is essential to confirm NHHR’s predictive value and its clinical utility in managing testosterone decline and TD.

## Data Availability

The original contributions presented in the study are included in the article/supplementary material. Further inquiries can be directed to the corresponding authors.
